# Transcriptome Analysis of a Spray Drying-Resistant Subpopulation Reveals a Zinc-Dependent Mechanism for Robustness in *L. lactis* SK11

**DOI:** 10.3389/fmicb.2018.02418

**Published:** 2018-10-15

**Authors:** Annereinou R. Dijkstra, Marjo J. C. Starrenburg, Tilman Todt, Sacha A. F. T. van Hijum, Jeroen Hugenholtz, Peter A. Bron

**Affiliations:** ^1^Kluyver Centre for Genomics of Industrial Fermentation, Delft, Netherlands; ^2^Nederlands Instituut Voor Zuivel Oonderzoek (NIZO), Ede, Netherlands; ^3^Swammerdam Institute for Life Sciences, Universiteit van Amsterdam, Amsterdam, Netherlands; ^4^Centre for Molecular and Biomolecular Informatics, Radboud umc, Nijmegen, Netherlands

**Keywords:** *Lactococcus lactis* SK11, robustness, spray drying, heat stress, transcriptome, zinc

## Abstract

The viability of starter cultures is essential for an adequate contribution to the fermentation process and end-product. Therefore, robustness during processing and storage is an important characteristic of starter culture strains. For instance, during spray drying cells are exposed to heat and oxidative stress, generally resulting in loss of viability. In this study, we exposed the industrially relevant but stress-sensitive *Lactococcus lactis* strain SK11 to two cycles of heat stress, with intermediate recovery and cultivation at moderate temperatures. After these two cycles of heat exposure, the abundance of robust derivatives was increased as compared with the original culture, which enabled isolation of heat-resistant subpopulations displaying up to 1,000-fold enhanced heat stress survival. Moreover, this heat-resistant subpopulation demonstrated an increased survival during spray drying. Derivatives from two independent lineages displayed different transcriptome changes as compared with the wild type strain, indicating that the increased robustness within these lineages was established by different adaptive strategies. Nevertheless, an overlap in differential gene expression in all five derivatives tested in both lineages included three genes in an operon involved in zinc transport. The link between zinc homeostasis and heat stress survival in *L. lactis* was experimentally established by culturing of the wild type strain SK11 in medium with various levels of zinc ions, which resulted in alterations in heat stress survival phenotypes. This study demonstrates that robust derivatives of a relatively sensitive *L. lactis* strain can be isolated by repeated exposure to heat stress. Moreover, this work demonstrates that transcriptome analysis of these robust derivatives can provide clues for improvement of the robustness of the original strain. This could boost the industrial application of strains with specific desirable traits but inadequate robustness characteristics.

## Introduction

Lactic acid bacteria (LAB) have been applied in food fermentation since ancient times for their role in preservation and for their flavor-enhancing properties (Leroy and De Vuyst, 2004). Since the start of industrial food fermentation, improvement of industrially relevant characteristics of the employed strains has been an ongoing process (Leroy and De Vuyst, 2004; Bachmann et al., 2014; Derkx et al., 2014; Dijkstra and Bron, 2018). Recently, interest has increased in the use of evolutionary engineering and selection strategies for strain improvement (Bachmann et al., 2014; Derkx et al., 2014). With these methods, strains with a specific desired phenotype are obtained by applying conditions in evolution and selection experiments that are advantageous for mutants with this phenotype and generally involve a higher growth rate or an increased ability to survive. The development of next-generation sequencing techniques has enabled the use of full genome resequencing and/or RNA sequencing for characterization of evolved or selected mutants to obtain further understanding of the mechanisms responsible for the desired traits (Bachmann et al., 2014; Derkx et al., 2014). In contrast to targeted mutagenesis the mutants obtained are considered to be natural, which enables their application in food fermentation without regulatory hurdles (Johansen, 2018).

One of the most extensively used lactic acid bacteria in food fermentation is *Lactococcus lactis*, notably in the production of cheese, butter, and buttermilk (Leroy and De Vuyst, 2004). Starter culture strains are generally selected for their flavor-forming and fast-acidifying characteristics, complemented with robustness characteristics during industrial processing (Smit et al., 2005; Bron and Kleerebezem, 2011; Papadimitriou et al., 2016).

Adaptive evolution and selection strategies have been successfully applied in *L. lactis* strains. For example, prolonged propagation in milk resulted in the adaptation of the plant-derived *L. lactis* strain KF147 to a dairy environment (Bachmann et al., 2012). Genome resequencing and comparative transcriptome analyses revealed changes in nitrogen metabolism and down-regulation of genes involved in degradation of plant material in the milk-adapted derivatives (Bachmann et al., 2012).

Changes in robustness of *L. lactis* strains have been achieved by targeted and random mutagenesis (Duwat et al., 1999; Rallu et al., 2000; Cesselin et al., 2009), but also spontaneous mutations have resulted in mutants with more robust phenotypes (Rochat et al., 2005; Smith et al., 2012). Alternatively, improved robustness of *L. lactis* strains can be realized by pre- or cross-adaptation, during which cells are subjected to a mild stress before exposure to the same or a different lethal stress, respectively. For example, previous studies demonstrated that a relatively high oxygen level during fermentation resulted in an increased robustness toward heat stress of *L. lactis* strain MG1363 and exposure to mild acid stress increased survival of strain NCDO712 during subsequent lethal doses of acid, heat, osmotic, oxidative, and ethanol stress (O'Sullivan and Condon, 1997; Dijkstra et al., 2014a). However, these adaptation methods can complicate the fermentation process, effects of adaptation conditions on robustness vary among strains (Dijkstra et al., 2016) and robustness is considered to be only improved for a short period in contrast to mutants obtained from evolution and selection experiments.

*L. lactis* subsp. *cremoris* strain SK11, which has been isolated from a cheese starter culture, has been previously demonstrated to be relatively sensitive toward heat stress and spray drying (Dijkstra et al., 2014b). Furthermore, heat stress survival of SK11 was shown to be highly influenced by fermentation conditions (Dijkstra et al., 2016). In this study, we repeatedly exposed strain SK11 to heat stress to select derivatives with improved robustness, which is relevant for survival during spray drying (Santivarangkna et al., 2008; Dijkstra et al., 2014b). The gene expression levels in the robust derivatives were compared to the wild type strain to gain insight in the molecular mechanisms driving improved robustness. This approach revealed a potential role for zinc in robustness of this strain which could be experimentally verified.

## Materials and methods

### Strains and growth conditions

*L. lactis* strain SK11 (Makarova et al., 2006) and derivatives were grown in M17 broth (Oxoid, Basingstoke, United Kingdom) supplemented with 0.5% (w/v) glucose (Merck, Darmstadt, Germany) (GM17) at 30°C. Selected colonies were incubated in 96-well plates (Greiner Bio-One, Germany) for 2 days and then supplemented with 30% glycerol and stored at −80°C.

### Selection experiment for robust derivatives

Strain SK11 was cultured at 30°C in 50 ml GM17. When stationary phase of growth was reached, two aliquots of 10 ml were collected and handled individually throughout the remainder of the experiments (lineage A and lineage B). Cells were centrifuged for 10 min at 1,865 × g and resuspended in 10 ml of 50 mM sodium phosphate (Merck) buffer pH 7.2 and transferred in 96 aliquots to 0.1 ml 96-well PCR plates to achieve a more consistent temperature profile in the entire sample during heat treatment (MicroAmp, Applied BioSystems, Foster City, USA). Heat stress was applied by incubation at 50°C for 40 min in a Gene-Amp PCR system 9700 (Applied BioSystems, Foster City, USA). After incubation, the aliquots were recombined and transferred back to 10 ml tubes and centrifuged for 10 min at 1,865 × g. Cells were resuspended in 10 ml GM17 and incubated at 30°C to allow growth of the remaining living cells. When these cells had reached stationary phase of growth (determined by pH-measurement), the above described procedure was repeated for both cultures. During each cycle, survival was determined by spotting serial dilutions of the samples before and after applying heat stress in triplicate on M17 agar plates supplemented with 0.5% glucose (Sieuwerts et al., 2008). Colony forming units (CFU) were determined after incubation of the plates for 3 days at 30°C.

### Heat stress survival assay

Heat stress survival was determined by a method previously developed in our laboratory, with minor modifications (Dijkstra et al., 2014b). Cells were harvested from 1 ml of culture in 96-deepwell plates (Greiner Bio-One) by centrifugation at 1,865 × g for 10 min and resuspended in 1 ml sterile 50 mM sodium phosphate (Merck) buffer pH 7.2. To measure heat stress survival, 0.1 ml of the cell suspensions were incubated at 50°C for 30 min in a 96-well PCR plate (MicroAmp, Applied BioSystems, Foster City, USA) in a Gene-Amp PCR system 9700 (Applied BioSystems, Foster City, USA). Survival was assessed by spotting 2 μl of serial dilutions with a Tecan Freedom Evo (Tecan, Switzerland) on M17 agar plates supplemented with 0.5% glucose. Colony forming units (CFU) were determined after incubation of the plates for 1–3 days at 30°C.

### Spray drying

Lab-scale spray drying was performed as described previously (Dijkstra et al., 2014b). Briefly, after overnight growth in 200 ml GM17, cells were harvested by centrifugation at 3,315 × g for 7 min and resuspended in 20% (w/v) skim milk powder. Cell suspensions were dried in a mini lab-scale spray dryer (model B-290, Büchi Labortechnik AG, Flawil, Switzerland) by using an inlet temperature of 200°C and an outlet temperature of 100°C. Ice water was continuously used to cool the nozzle. The generated powders were rehydrated [1% (w/v)] in a solution containing peptone (1 g/l) and sodium chloride (8.5 g/l) and incubated at room temperature for 30–60 min. To determine survival, the rehydrated cell suspension and the feed cell suspension were serially diluted in duplicate and spotted (5 μl) in triplicate on GM17 agar plates (Sieuwerts et al., 2008). After 3 days of incubation at 30°C, colony forming units were determined. Dry weight of the cell suspensions was assessed in triplicate by measuring the weight of 5 ml of sample after incubation at 55°C for 5 days.

### RNA isolation and sequencing

For transcriptome analysis, SK11 and its robust derivatives A3, A5, B1, B2, and B7 were cultured in duplicate in 50 ml GM17 at 30°C. In exponential phase of growth (OD 0.8–1.1) samples were taken for RNA isolation. RNA isolation was performed using routine procedures, as described previously (Bron et al., 2012) with minor adjustments. Aliquots of 5 ml of culture were centrifuged at 4,000 × g for 3 min at 2°C and cells were resuspended in 0.5 ml cold TE buffer. To this suspension, 500 μl 1:1 phenol/chloroform, 30 μl 10% SDS, 30 μl 3M sodium acetate pH 5.2 and 500 mg 0.1 mm zirconia beads (Biospec Products, Inc., Bartlesville, USA) was added in a 2 ml screw-cap tube and samples were frozen in liquid nitrogen and stored at −80°C. cDNA synthesis and sequencing with an Ion Proton system using an Ion PI-chip (Life Technologies) was performed by PrimBio Research Institute (Exton, USA). The data have been deposited in NCBI's Gene Expression Omnibus and are accessible through GEO Series Accession number GSE119647.

### Data analysis

Demultiplexed fastq-files were analyzed using Transcriptor with default settings (Todt et al., submitted for publication). Read data were mapped against the reference genome sequence of SK11 using Bowtie2 (Langmead and Salzberg, 2012). Transcripts were determined by applying a segmentation algorithm to the alignment results (Todt et al., 2012). Segments with read coverage above a minimum read count threshold of five reads were identified and adjacent segments were joined. For each sample, transcripts were annotated by matching them with gene annotations provided by the selected reference genome. Read counts were used to identify differentially expressed genes across the different derivatives and the original SK11 strain using edgeR (Robinson et al., 2010). *P*-values were corrected for multiple testing using the Benjamini and Hochberg procedure provided by the edgeR package. We selected the genes with a false discovery rate < 0.05 and an absolute log2 fold change >2 for comparison of the robust derivatives and the original SK11 strain for further analysis. Multivariate Redundancy Analysis was done using Canoco 5.04 (Ter Braak and Smilauer, 2012), using default settings of the analysis type “Constrained.” Z-transformed RNA expression values (for each sample average 0, standard deviation 1) for the genes was used as response data and the sample lineages as explanatory variable.

### Assessment of the effect of zinc on robustness

The effect of zinc on the heat stress survival of strain SK11 and the robust derivatives was assessed by growing the strains in chemically defined medium (CDM). The composition of CDM was as described previously (Wegkamp et al., 2007; Dijkstra et al., 2014a) with varying concentrations of zinc (0, 0.017, 0.17, 1.7, 17, or 174 μM). Heat stress survival was assessed as described above, with the minor adjustment that the cells were exposed to 50°C for 20 min.

## Results

### Repeated exposure to heat stress results in enrichment of robust derivatives

**Figure 1:**
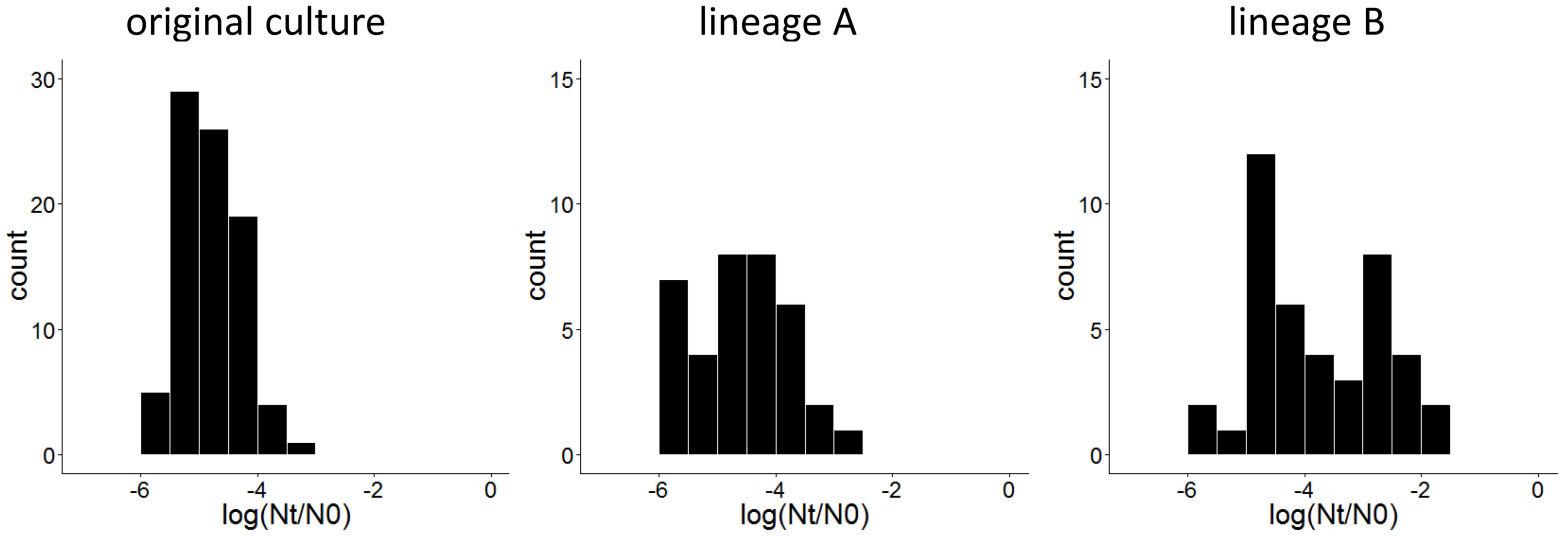
Distribution of survival after 30 min at 50°C, expressed as the difference between log CFU/ml after stress (Nt) and before stress (N0) of 84 isolates of the original culture and 36 and 42 isolates after two cycles of heat stress from lineage A and lineage B, respectively.

To obtain derivatives of *L. lactis* SK11 with increased robustness toward spray drying, cell suspensions (two independent replicates) were exposed twice to heat stress with intermediate liquid culturing of the surviving subpopulation. After both cycles of stress, a similar survival of the population (±0.003%) was observed. However, comparing individual colonies of surviving cells after the second cycle of heat stress with individual colonies from the original culture revealed an increase in robust variants in both lineages (Figure 1). Although both lineages were processed identically, lineage B included isolates with a higher robustness than those from lineage A (Figure 1).

**Figure 2:**
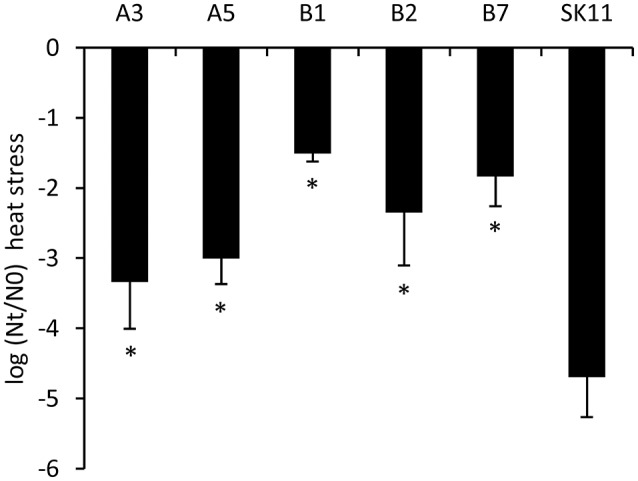
Survival during heat stress of the original strain SK11 and five heat-robust derivatives, expressed as the difference in log CFU/ml (heat stress) after stress (Nt) and before stress (N0). The data represent averages of four biological replicates. Error bars indicate standard deviations. ^*^P < 0.05 (*t*-test, compared with SK11).

To determine the stability of the robustness phenotype, the 10 most robust isolates obtained after two cycles of stress from each lineage were cultured for ~20 generations. Subsequent reassessment of their robustness toward heat stress revealed that highly improved robustness phenotypes were still present. From these cultures, we selected the most robust isolates from each lineage (A3 and A5 from lineage A and B1, B2, and B7 from lineage B), which displayed a 25 to over 1,000 times increased survival as compared with the original strain (Figure 2).

These data demonstrate that by repeated exposure to heat stress, more robust derivatives can be enriched within a population, which enables the isolation of these more robust derivatives. Moreover, we demonstrated that the improved robustness toward heat stress is a stable characteristic upon culturing of these derivatives.

### Heat-robust derivatives display enhanced spray drying survival

**Figure 3:**
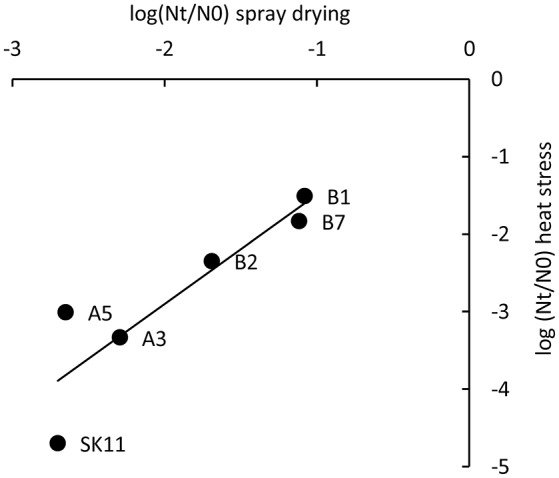
Correlation of survival during heat stress and during spray drying of SK11 and five robust derivatives, expressed as the difference in log CFU/ml (heat stress) or CFU/g (spray drying) after stress (Nt) and before stress (N0). The data represent averages of four (heat stress) or two (spray drying) biological replicates. Pearson correlation coefficient = 0.89.

To study if the five robust derivatives A3, A5, B1, B2, and B7 were also more robust toward spray drying as compared with the original strain SK11, all strains were dried in a lab-scale spray dryer. Derivative B1 displayed the smallest loss in viability during spray drying (20% survival) as compared with SK11 (0.5% survival), whereas the other derivatives displayed survival rates ranging from 0.6 (A5) to 19% (B7). These observations are in line with the fact that derivative B1 also displayed the smallest loss in viability during heat stress. Concomitantly, a significant correlation between heat stress survival and spray drying survival of the derivatives and the original strain was observed (Pearson correlation coefficient = 0.89, Figure 3). Taken together, these data establish that derivatives with an improved survival during spray drying can be obtained by repeated exposure to heat stress.

### Transcriptome analysis associates zinc transport with robustness

To better understand the increase in robustness of the robust derivatives, we performed an RNAseq-based transcriptome analysis (Supplementary data). The five derivatives and the original strain SK11 were cultured in GM17 and cells were harvested from exponential phase of growth for RNA isolation and assessment of heat stress survival. Similar to what we observed in stationary phase of growth, all derivatives displayed a significantly increased robustness toward heat stress in the exponential phase of growth as compared with the original strain SK11 (data not shown).

**Figure 4:**
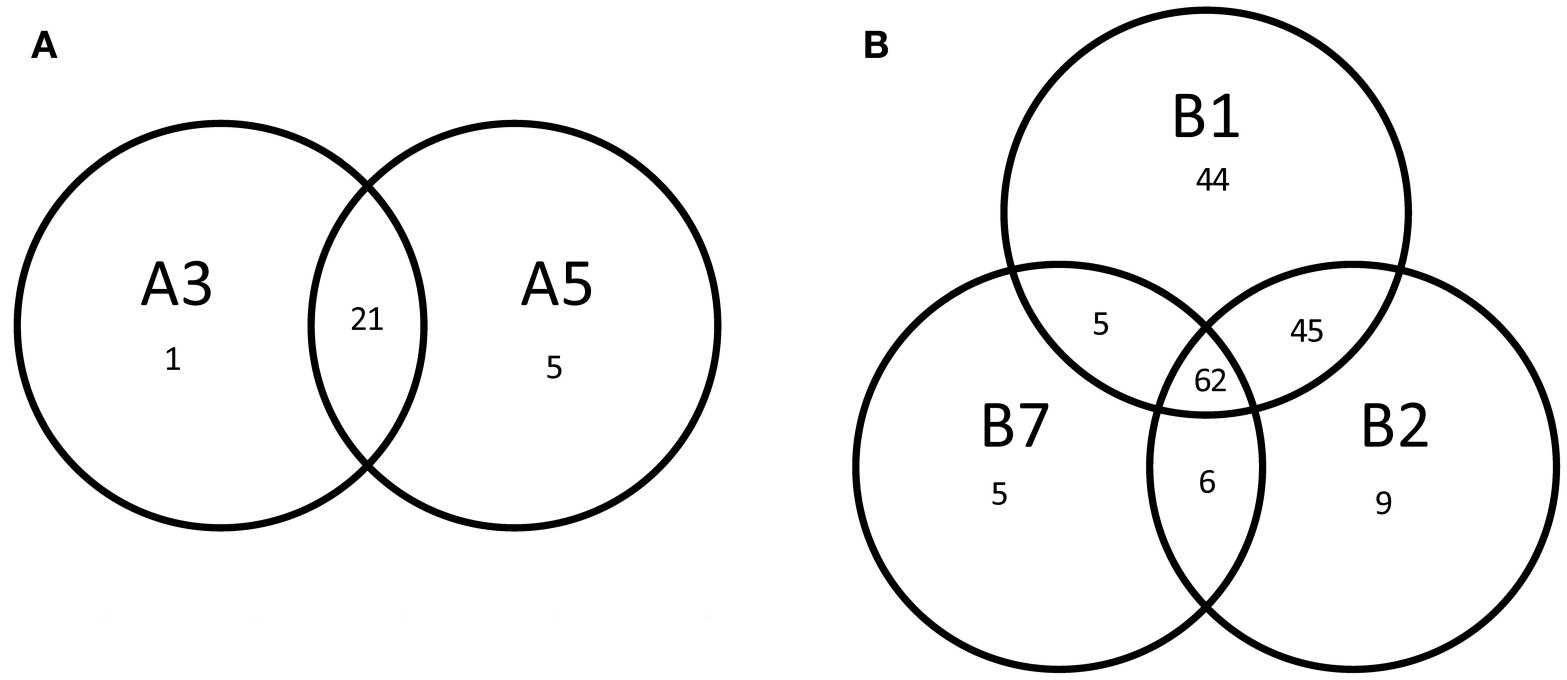
Venn diagrams of the number of differentially expressed genes (fdr < 0.05, absolute (log2 FC) > 2) of the robust derivatives from lineage **(A)** and lineage **(B)** as compared with the original strain SK11.

Multivariate Redundancy Analysis demonstrated that there was no significant difference between the transcriptomes of each replicate, whereas transcriptomes of both lineages and wild-type could be readily distinguished from each other (*P* = 0.002). Moreover, it revealed a significant difference between the transcript levels of derivatives from lineage A and derivatives from lineage B (*P* = 0.004), suggesting that the underlying molecular mechanism resulting in improved robustness differed between the lineages. We selected the genes with a significantly different gene expression level as compared with the original strain SK11 (fdr < 0.05 and absolute(log2 FC) > 2) for further analysis. The derivatives within each lineage displayed a high similarity in differentially expressed genes (Figure 4). The transcriptome of derivative B1 (156 differentially expressed genes) displayed the most changes as compared with SK11 (Figure 4), which might explain the large increase in robustness that was observed for this derivative (Figure 2). Notably, the majority of the genes that were only significantly differentially expressed in B1 and/or B2 were similarly regulated in the other derivative(s) from lineage B, but with a fold change below cutoff.

An overlap of 21 significantly differentially expressed genes as compared with SK11 was found in the derivatives from lineage A, including genes encoding a glycosyltransferase and an outer membrane protein as well as multiple genes encoding transposases (Figure 4 and Table 1). Also two genes that are considered pseudogenes, a partial glycosyltransferase encoding gene (*LACR_1378*) and a partial UDP-*N*-acetylglucosamine 2-epimerase encoding gene (*LACR_1381*) were upregulated in the robust derivatives of lineage A. In the group of derivatives from lineage B 62 genes were differently expressed, including genes involved in maltose and pyrimidine metabolism (Figure 4 and Table 1). Interestingly, four genes were differently expressed in all derivatives as compared with the original SK11 strain (Table 1). These included three genes from an operon containing genes involved in zinc transport (*LACR_2417, LACR_2419*, and *LACR_2420*), which were downregulated in the derivatives. Notably, the fourth gene present in this operon (*LACR_2418*) displayed a significant lower expression in all derivatives as well, but for derivative A5 the fold change was slightly below cutoff (Supplementary data). Furthermore, a gene encoding a hypothetical protein (*LACR_0948*) displayed an increased expression in the robust derivatives compared with SK11. Further *in silico* analyses revealed that this protein is highly similar to a cell division protein in another *L. lactis* strain (TIFN6).

**Table 1:** Differentially expressed genes in the robust derivatives of lineages A and B compared with SK11.

**Locus tag**	**gene**	**Function**	**fc A3**	**fdr A3**	**fc A5**	**fdr A5**
**ROBUST DERIVATIVES FROM REPLICATE A**						
*LACR_0949*		Glycosyltransferase	7.17	1.10^−90^	6.84	7 × 10^−83^
*LACR_0149*		Outer membrane protein	5.45	6.10^−55^	5.70	3.10^−59^
*LACR_0948*		Hypothetical_protein	8.45	2.10^−60^	8.13	7.10^−55^
*LACR_0416*		Transposase	−4.34	6.10^−44^	−4.60	5.10^−49^
*LACR_1381*		Pseudogene	4.65	2.10^−30^	4.38	5.10^−27^
*LACR_2420*		Transcriptional regulator	−2.54	6.10^−26^	−2.98	2.10^−34^
*LACR_0882*		Transposase	−3.97	3.10^−26^	−3.77	6.10^−25^
*LACR_2419*		Zinc ABC transporter substrate-binding protein	−2.51	3.10^−20^	−3.05	2.10^−28^
*LACR_1441*		Hypothetical_protein	2.59	2.10^−23^	2.29	2.10^−18^
*LACR_2417*		Zinc ABC transporter permease	−2.26	2.10^−18^	−2.19	1.10^−17^
*LACR_0961*		Transposase	−3.74	8.10^−23^	−3.36	2.10^−19^
*LACR_1378*		Pseudogene	3.72	3.10^−21^	3.53	3.10^−19^
*LACR_0662*		Transposase	−3.43	2.10^−21^	−2.80	9.10^−17^
*LACR_1858*		Amidase	−2.93	3.10^−19^	−2.78	4.10^−18^
*LACR_1085*		Transposase	−2.80	1.10^−15^	−2.58	2.10^−14^
*LACR_1084*		Transposase	−3.11	3.10^−14^	−3.17	2.10^−15^
*LACR_0661*		Transposase	−3.04	1.10^−13^	−3.52	2.10^−17^
*LACR_1670*		Transposase	−2.49	3.10^−13^	−2.45	2.10^−13^
*LACR_1034*		Transposase	−2.98	2.10^−13^	−3.20	1.10^−15^
*LACR_1669*		Transposase	−3.15	1.10^−12^	−2.85	1.10^−11^
*LACR_1035*		Transposase	−2.24	3.10^−11^	−2.37	6.10^−13^
**Locus tag**	**gene**	**Function**	**fc B1**	**fdr B1**	**fc B2**	**fdr B2**	**fc B7**	**fdr B7**
**ROBUST DERIVATIVES FROM REPLICATE B**								
*LACR_1855*		ABC-type maltose transport system, permease component	−5.56	5.10^−65^	−4.96	1.10^−57^	−4.00	5.10^−45^
*LACR_0940*		ABC transporter permease	5.97	8.10^−51^	7.45	6.10^−70^	4.92	1.10^−37^
*LACR_1847*		maltose phosphorylase	−5.90	6.10^−63^	−4.57	1.10^−46^	−4.61	2.10^−47^
*LACR_0941*		Peptide ABC transporter ATPase	6.08	1.10^−49^	7.41	2.10^−66^	5.02	2.10^−36^
*LACR_0535*		YG repeat-containing cell wall-associated hydrolase	3.57	9.10^−26^	6.57	3.10^−81^	3.99	5.10^−32^
*LACR_2417*		zinc ABC transporter permease	−3.92	5.10^−46^	−3.81	6.10^−44^	−3.28	2.10^−35^
**LACR_2420**		Transcriptional regulator	−3.23	1.10^−39^	−3.24	2.10^−39^	−3.45	5.10^−44^
*LACR_0952*		Nicotinamide mononucleotide transporter	−2.70	7.10^−29^	−3.23	2.10^−38^	−3.57	6.10^−45^
*LACR_2086*		Glycosyltransferase	−3.76	3.10^−41^	−3.73	2.10^−40^	−2.58	2.10^−23^
*LACR_2419*		Zinc ABC transporter substrate-binding protein	−3.40	9.10^−35^	−3.35	1.10^−33^	−3.39	2.10^−34^
*LACR_1848*		Alpha-glucosidase	−5.84	3.10^−41^	−4.50	8.10^−31^	−4.12	5.10^−28^
*LACR_1854*		ABC-type sugar transport system, permease component	−4.83	4.10^−39^	−4.58	2.10^−36^	−3.17	3.10^−22^
*LACR_2418*		Zinc ABC transporter ATP-binding protein	−3.79	9.10^−38^	−3.49	3.10^−33^	−2.98	4.10^−26^
*LACR_0249*		HAD superfamily hydrolase	5.05	3.10^−39^	4.53	3.10^−33^	3.52	1.10^−21^
*LACR_2087*		Hypothetical protein	−3.07	1.10^−32^	−3.53	8.10^−40^	−2.24	2.10^−19^
*LACR_1849*		Alpha-amylase	−5.04	3.10^−37^	−3.90	6.10^−27^	−3.70	2.10^−25^
*LACR_0939*		Hypothetical protein	6.35	1.10^−28^	7.96	6.10^−41^	5.12	2.10^−19^
*LACR_1850*		Maltose O-acetyltransferase	−5.87	2.10^−37^	−4.30	3.10^−27^	−3.43	1.10^−20^
*LACR_0646*		Hypothetical protein	3.40	4.10^−32^	3.63	6.10^−36^	2.22	5.10^−15^
*LACR_1852*		Neopullulanase	−3.98	5.10^−41^	−2.72	8.10^−23^	−2.39	2.10^−18^
*LACR_1853*		Maltose ABC transporter substrate binding protein	−4.59	7.10^−33^	−4.31	9.10^−30^	−2.98	2.10^−16^
*LACR_1201*		Hypothetical protein	4.41	8.10^−28^	5.18	6.10^−36^	2.67	9.10^−12^
*LACR_1335*		ATP phosphoribosyltransferase regulatory subunit	3.92	4.10^−30^	3.33	5.10^−22^	3.04	2.10^−18^
*LACR_0895*	*mnmA*	tRNA-specific 2-thiouridylase MnmA	−2.55	3.10^−25^	−2.37	5.10^−22^	−2.01	3.10^−16^
*LACR_0339*		Hypothetical protein	8.10	1.10^−23^	8.98	2.10^−28^	5.60	7.10^−11^
*LACR_1851*		Trehalose-6-phosphate hydrolase	−5.08	4.10^−28^	−3.46	9.10^−17^	−3.19	7.10^−15^
*LACR_1159*	*pyrE*	Orotate phosphoribosyltransferase	3.30	7.10^−23^	3.15	3.10^−21^	2.42	3.10^−13^
*LACR_1498*	*carB*	Carbamoyl phosphate synthase large subunit	2.88	5.10^−20^	2.73	3.10^−18^	2.40	2.10^−14^
*LACR_1712*		Bifunctional pyrimidine regulatory protein PyrR uracil phosphoribosyltransferase	3.16	1.10^−22^	2.87	3.10^−19^	2.01	3.10^−10^
*LACR_1726*		Subtilisin-like serine protease	4.66	3.10^−21^	4.50	8.10^−20^	3.24	3.10^−10^
*LACR_1160*	*pyrC*	Dihydroorotase	3.03	2.10^−19^	2.87	1.10^−17^	2.30	6.10^−12^
*LACR_1470*		Dihydroorotate oxidase B, electron transfer subunit	2.79	1.10^−18^	2.81	7.10^−19^	2.10	3.10^−11^
*LACR_1469*		dihydroorotate dehydrogenase 1B	2.74	2.10^−17^	2.77	1.10^−17^	2.31	1.10^−12^
*LACR_0161*		Major facilitator superfamily permease	−2.40	5.10^−17^	−2.40	9.10^−17^	−2.06	4.10^−13^
*LACR_1332*		Histidinol dehydrogenase	3.83	6.10^−18^	3.49	5.10^−15^	3.29	2.10^−13^
*LACR_1711*		Xanthine/uracil permease	2.87	2.10^−19^	2.61	2.10^−16^	2.02	2.10^−10^
*LACR_0229*		Hypothetical protein	3.95	9.10^−13^	5.54	3.10^−25^	2.96	6.10^−07^
*LACR_1368*		5-methyltetrahydropteroyltriglutamate homocysteine S-methyltransferase	2.09	1.10^−08^	3.50	2.10^−21^	2.83	2.10^−14^
*LACR_1995*		Amino acid transporter	−2.62	4.10^−16^	−2.32	3.10^−13^	−2.51	6.10^−15^
*LACR_0240*		NADPH:quinone reductase related Zn-dependent oxidoreductase	3.04	6.10^−15^	3.12	1.10^−15^	2.83	6.10^−13^
*LACR_2561*		Metal-dependent membrane protease	3.00	1.10^−16^	2.53	5.10^−12^	2.80	2.10^−14^
*LACR_1334*		Hypothetical protein	4.13	8.10^−17^	3.72	1.10^−13^	3.51	6.10^−12^
*LACR_1467*		Hypothetical protein	2.46	1.10^−14^	2.39	8.10^−14^	2.14	3.10^−11^
*LACR_1710*	*pyrB*	aspartate carbamoyltransferase catalytic subunit	2.82	4.10^−16^	2.63	3.10^−14^	2.05	3.10^−09^
*LACR_0241*		Nucleoside-diphosphate sugar epimerase	3.19	7.10^−13^	3.49	8.10^−15^	2.93	8.10^−11^
*LACR_0242*		Saccharopine dehydrogenase related protein	3.30	4.10^−13^	3.48	3.10^−14^	2.77	1.10^−09^
*LACR_1309*		Ketol-acid reductoisomerase	2.33	2.10^−13^	2.19	6.10^−12^	2.01	5.10^−10^
*LACR_2542*		Hypothetical protein	−3.30	9.10^−14^	−3.27	3.10^−13^	−2.27	7.10^−08^
*LACR_1327*		Imidazole glycerol phosphate synthase subunit HisF	3.42	2.10^−12^	3.23	4.10^−11^	3.14	3.10^−10^
*LACR_1709*		Carbamoyl phosphate synthase small subunit	2.72	1.10^−13^	2.46	2.10^−11^	2.02	4.10^−08^
*LACR_0847*		Hypothetical protein	−2.24	9.10^−12^	−2.24	1.10^−11^	−2.06	6.10^−10^
*LACR_1468*		Orotidine 5'-phosphate decarboxylase	2.54	2.10^−12^	2.45	1.10^−11^	2.12	6.10^−09^
*LACR_1161*		TPR repeat-containing protein	5.65	1.10^−14^	5.31	2.10^−13^	2.70	1.10^−04^
*LACR_1539*		Transcription regulator	3.21	3.10^−16^	2.18	2.10^−08^	2.10	9.10^−08^
*LACR_1333*		Pseudogene	3.18	1.10^−11^	3.03	2.10^−10^	2.75	1.10^−08^
*LACR_0514*		Hypothetical protein	−2.78	5.10^−12^	−2.40	2.10^−09^	−2.08	2.10^−07^
*LACR_1336*		Histidinol-phosphate aminotransferase	3.53	8.10^−13^	3.14	3.10^−10^	2.23	2.10^−05^
*LACR_2541*		Universal stress protein UspA-like nucleotide-binding protein	−3.85	2.10^−10^	−3.53	6.10^−09^	−2.43	3.10^−05^
*LACR_1699*		Ammonia permease	−2.80	7.10^−09^	−2.13	4.10^−06^	−2.90	3.10^−09^
*LACR_2540*		Major facilitator superfamily permease	−3.23	6.10^−08^	−3.69	3.10^−09^	−2.69	8.10^−06^
*LACR_0127*		Major facilitator superfamily permease	2.61	4.10^−07^	2.03	9.10^−05^	2.01	1.10^−04^
*LACR_0948*		Hypothetical protein	2.50	1.10^−02^	4.67	3.10^−11^	2.22	4.10^−02^

*Overlapping genes are underlined. Log2 fold changes (fc) and false discovery rates (fdr) are displayed of each of the derivatives from lineage A or B compared with SK11. A negative or positive fc indicates that the gene was downregulated or upregulated, respectively, in the robust derivative*.

Overall, it was demonstrated that the heat-robust derivatives from each lineage display different changes in gene expression compared with the original strain. Besides the differences in transcript levels between the derivatives of both lineages, genes involved in zinc transport were shown to be downregulated in all derivatives, which strongly suggests that these genes are involved in increased robustness toward heat stress and consequently spray drying.

### Presence of zinc during growth improves heat stress survival of SK11

**Figure 5:**
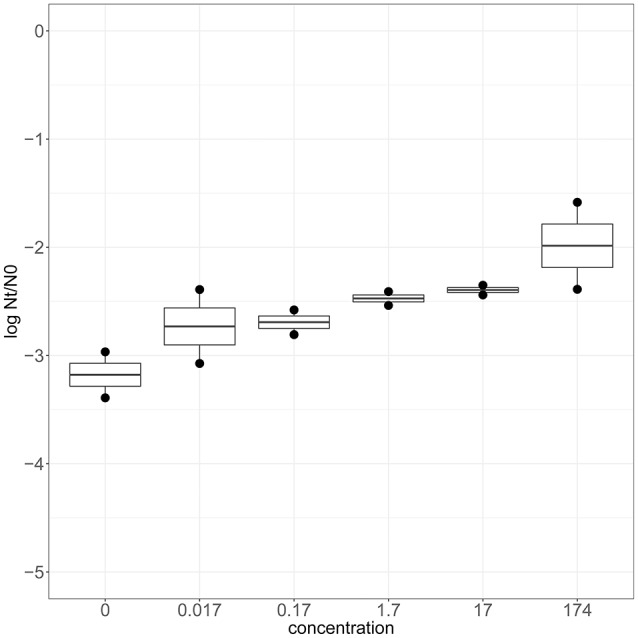
Heat stress survival of SK11 after culturing at various concentrations of Zn^2+^ expressed as the difference in log CFU/ml after stress (Nt) and before stress (N0). The data represent averages of two biological replicates.

To confirm that zinc is involved in robustness toward heat stress, wild type strain SK11 was cultured in CDM with various levels of zinc (0–174 μM). Adjustment of the zinc concentration in the culture medium did not significantly affect the growth rate (0.60 ± 0.04 h^−1^) or final optical density (0.64 ± 0.04) of SK11. When cultured at relatively high zinc levels SK11 displayed a higher survival after exposure to heat stress (up to 10 times) than when cultured at lower zinc levels (Figure 5). These data demonstrate that transcriptomic analysis of selected robust derivatives is useful to provide clues to improve robustness of the original strain and, moreover, confirms that a link between zinc homeostasis and heat stress survival exists in *L. lactis* SK11.

## Discussion

Strains with specific desirable traits might not be suitable for application in starter cultures due to poor survival characteristics during spray drying prior to their employment in food matrices. We demonstrated that robust derivatives of a spray drying-sensitive strain can be isolated after repeated exposure to heat stress which concomitantly displayed up to a more than 40 times increased survival during spray drying. The fact that these robust phenotypes appeared stable upon culturing strongly suggests that these phenotypes are due to (a) genetic mutation(s), resulting in transcriptome changes. As it is expected that our heat stress assay is too severe to allow adaptation of cells, either by genetic mutation or regulatory switches, it seems likely that the heat-robust derivatives already exist in the starting population (10^10^ cells) due to spontaneous genetic variation (Barrick and Lenski, 2013). Two cycles of heat stress increased the abundance of robust derivatives to a level that enabled selection from a relatively small set of isolates which was not feasible from the original population.

As expected, genome resequencing revealed only a few single-nucleotide polymorphisms (SNPs) and small deletions in the chromosomes of the robust derivatives (Supplementary data 2). Manual inspection did not reveal any direct link to genes that were demonstrated to be up- or downregulated by RNAseq-based transcriptome analysis, which could suggest that the robust phenotype is rather complex. However, each derivative of lineage B had a distinct mutation in the operon encoding the two-component system KinF/LlrF (B1: *LACR_1844*-V171G; B2: *LACR_1845*-D177Y; B7: *LACR_1845*-F21L). Little appears known about the exact functions of this operon, but it has been previously linked to oxidative stress survival in *L. lactis* strain MG1363 (O'Connell-Motherway et al., 2000). A link with heat stress survival or zinc homeostasis has, to the best of our knowledge, not been established. The latter correlation appears unlikely anyway because this mutation was only found in the derivatives of lineage B, whereas the zinc operon was down-regulated in the derivatives of both lineage A and B. The mutation might alter the activity of the regulator encoded which might impact on its regulon which could include the regulated genes identified in the transcriptome analyses of lineage B.

If desirable traits of the strain are not necessarily required to survive during the selection experiment, these characteristics might be lost. The derivatives from lineage B displayed a normal growth in milk, whereas the derivatives from lineage A displayed a reduced growth in milk as compared with the original strain (data not shown). Selection pressure on specific traits (if possible) during the intermediate culturing step of a selection experiment might prevent loss of these traits. The culturing step in between the cycles of heat stress was anticipated to select for robust derivatives with an adequate growth rate. Some of the robust derivatives (A3 and A5) displayed a similar maximum growth rate as the original strain, but other derivatives (B1, B2, and B7) displayed a decreased maximum growth rate (data not shown). The fact that these could still be enriched despite the apparent counter selection in the intermediate culturing step showcases the dramatically increased robustness phenotypes that are present in small subpopulations of cells in a culture. Moreover, no significant correlation was observed between growth rate and stress survival of the isolates after two cycles of heat stress, demonstrating that an increase in robustness can be achieved without necessarily reducing the growth rate, as has been previously demonstrated in multiple *L. lactis* strains as well (Dijkstra et al., 2014a, 2016).

The transcriptomes of the derivatives within each lineage were highly similar, whereas between the lineages the set of genes displaying altered expression as compared with the original strain were significantly different. Together with the observed differences in robustness phenotypes and growth rates of the derivatives this strongly indicates that different mechanisms resulted in increased robustness toward heat stress in each lineage.

Two genes, encoding a HAD superfamily hydrolase (*LACR_0249*) and a hypothetical protein (*LACR_1467*), that were differentially expressed in the derivatives of lineage B, were previously associated with heat stress survival in SK11, which supports the suggested role of these genes in robustness (Dijkstra et al., 2016). Besides these two genes, no overlap was observed between the transcriptome signature associated with heat stress survival (124 genes) that we previously reported for SK11 (Dijkstra et al., 2016) and the transcriptome changes revealed in the heat-robust derivatives in this study. This further illustrates the diversity in survival mechanisms within this strain and suggests that the transcriptome changes that are induced in the heat-robust derivatives were not induced by the fermentation parameters (salt, oxygen, pH, temperature) as applied in our previous study or other survival mechanisms had a more pronounced effect in the observed differences in robustness phenotypes (Dijkstra et al., 2016).

Besides the differences between the derivatives of both lineages also an overlap in differentially expressed genes was observed. Four genes, including three genes involved in zinc transport, were observed to be differentially expressed in all five derivatives as compared with the original strain, which strongly suggests involvement of these genes in robustness toward heat stress. Unfortunately, strain SK11 is not genetically accessible to the extent that routine strategies can be employed to construct gene deletion mutants. Moreover, our earlier work (Dijkstra et al., 2016) shows that distinctly different stress responses are induced in different *L. lactis* strains, preventing the employment of a model strain for gene deletions. Therefore, to validate that zinc transport is connected to heat stress survival, the wild type strain SK11 was cultured in presence of various levels of zinc which resulted in differences in heat stress robustness phenotypes. The down-regulation of the zinc operon in the more robust derivatives appears not to correspond with the increased survival at higher zinc concentration of the original strain. One reason for the decreased expression might be that the robust derivatives already contained a higher amount of zinc. Moreover, it is unestablished whether this transporter imports or exports zinc.

Zinc has previously been suggested to be involved in oxidative stress survival in *L. lactis* subsp. *cremoris* strain MG1363 (Scott et al., 2000). More importantly, one of the genes of this zinc transport operon (*L167426*/*zitS*, an ortholog of *LACR_2419*) was associated with heat stress survival in IL1403 in an earlier study (Dijkstra et al., 2016).

Metal homeostasis in general has been linked to heat stress survival in various bacterial species and mostly involves divalent cations. For instance, O'Connor et al. demonstrated an increased thermotolerance of *Salmonella enterica* due to an increased expression of a gene encoding a Mg^2+^ transport protein (O'Connor et al., 2009). Heat shock in *Bacillus licheniformis* stimulated the transcription of iron uptake genes (Nielsen et al., 2010), whereas several genes encoding metal transporters were differently expressed at high temperature in *Staphylococcus aureus* (Fleury et al., 2009). We previously demonstrated the correlation between the presence of a manganese transporter and robustness toward heat stress in an *L. lactis* strain collection, whereas the expression of genes involved in iron transport associated with heat stress survival in strain MG1363 (Dijkstra et al., 2014a,b). In addition, heat stress survival of strain SK11 was associated with expression of a manganese transporter and a manganese dependent transcriptional regulator (Dijkstra et al., 2016). It is likely that the mentioned transporters are not all correctly annotated for their substrate and/or have side activities for the transport of other divalent metal cations. Therefore, all these observations could point toward a similar mechanism as observed in this study in which we demonstrate that there is a connection between zinc uptake and heat stress survival in *L. lactis*.

Taken together, our data demonstrate that repeated exposure to heat stress can increase the abundance of heat-robust derivatives of a strain. By isolating heat-robust derivatives of a strain the yield after spray drying can be increased dramatically (from 0.5 to 20 %). Furthermore, it was shown that transcriptomic analysis of robust derivatives can identify conditions resulting in improved robustness of the original strain. Moreover, a link between zinc homeostasis and heat stress survival in *L. lactis* was revealed. Overall, this could support the application of strains with specific desirable traits but inadequate robustness characteristics, broadening the set of strains with optimal industrial applicability.

## Author contributions

AD, JH, PB designed the experiments. AD, MS performed all the experiments. AD, TT, SH, JH, PB analyzed the data. AD, JH, PB drafted the manuscript. SH critically revised the manuscript. All authors read and approved the manuscript.

### Conflict of interest statement

AD, MS, SH and PB were employed by company NIZO food research B.V., The Netherlands. The remaining authors declare that the research was conducted in the absence of any commercial or financial relationships that could be construed as a potential conflict of interest.
